# Prognostic factors for the long term outcome after surgical celiac artery decompression in MALS

**DOI:** 10.1186/s13023-023-02952-7

**Published:** 2023-10-23

**Authors:** Anna Woestemeier, Alexander Semaan, Andreas Block, Jan Arensmeyer, Jonas Dohmen, Alexander Kania, Frauke Verrel, Martin Mücke, Jörg C. Kalff, Philipp Lingohr

**Affiliations:** 1 Department for General, Visceral, Thoracic and Vascular Surgery, University Hospital of Bonn, Bonn, Germany; 2 Institute for Digitalization and General Medicine, University Hospital Aachen, Aachen, Germany; 3 Center for Rare Diseases Aachen (ZSEA), University Hospital Aachen, Aachen, Germany

**Keywords:** Median arcuate ligament syndrome, Dunbar syndrome, Celiac artery compression syndrome, Mast cell activation syndrome, CT angiography, Vascular compression

## Abstract

**Background:**

The median arcuate ligament syndrome (MALS) is a rare disease caused by compression of the celiac artery (ORPHA: 293208). Surgical treatment of MALS aims to restore normal celiac blood flow by laparoscopic celiac artery decompression. However, surgical success rates vary widely between patients, therefore adequate selection of patients is essential to improve surgical outcome. Symptoms of MALS might also overlap with other chronic multi-system disorders such as mast cell activation syndrome (MCAS). So far, no clinical or radiological parameter was found to be predictive of the postoperative outcome. We, therefore, aim to study preclinical parameters in one of the largest MALS cohorts with the focus to identify patients that would benefit from surgical MAL release.

**Results:**

By analyzing 20 MALS patients that underwent surgical celiac artery decompression, we found 60% of patients (12/20) had a postoperative relief of their symptoms and a simultaneous decrease of analgetic use. No demographic, radiologic or operative parameter was able to predict postoperative symptom relief. However, mast cell activation syndrome correlated significantly (p = 0.04) with persistent symptoms after the operation.

**Conclusions:**

Overall, laparoscopic MAL release can provide immediate symptomatic relief. Despite the missing predictive value of demographic and imaging data, our data show a correlation between persistent symptoms and a co-existing mast cell activation syndrome. This suggests that MCAS symptoms might be interpreted as MALS symptoms in the presence of celiac artery stenosis and therefore surgical treatment should be evaluated carefully. Overall, the selection of patients who are most likely to respond to surgical MAL release may best be accomplished by an interdisciplinary team of gastroenterologists, radiologists and surgeons.

**Supplementary Information:**

The online version contains supplementary material available at 10.1186/s13023-023-02952-7.

## Introduction

Median arcuate ligament syndrome (MALS), also known as celiac artery compression syndrome, results from the extrinsic compression of the coeliac trunk by fibers of the median arcuate ligament [1]. The vascular compression causes nonspecific symptoms including nausea, vomiting, weight loss and postprandial epigastric pain. This rare syndrome (ORPHA: 293208) was first described in the early 1970s by Harjola and Dunbar who also reported the first case series involving surgical treatment of MALS [2, 3].

Pathophysiology of MALS is incompletely understood but may be related to both ischemic and neuropathic mechanisms. Increased demand for blood flow through a compressed celiac artery leads to foregut ischemia resulting in epigastric pain, although development of collateral vessels usually prevents the development of ischemia [4]. The neuropathic component may result from a combination of chronic compression and overstimulation of the celiac ganglion. This neuropathic compression is thought to lead to direct irritation of sympathetic pain fibers and splanchnic vasoconstriction and ischemia [5, 6].

MALS diagnostic workup is not standardized due to its rare occurrence leading to long latency periods in most patients. Usually more common causes are excluded by a myriad of (invasive) procedures including esophagogastroduodenoscopy, colonoscopy and cross sectional imaging [4, 7]. Symptoms of MALS might also overlap those of mast cell activation syndrome (MCAS). MCAS, a variant of primary mast cell activation diseases, is a clinically heterogeneous disease, whose pathology and etiology is still not understood. Consequently, this leads to difficulty in determining an accurate diagnostic process. According to current knowledge, MCAS is most likely the result of a variety of possible mutations. For instance, mutations in kinases, receptors and proteins of signal transduction inducing pathologically activated mast cells in different organ systems [8]. In contrast to systemic mastocytosis, an activating KIT point mutation in codon 816 is absent [9].

MCAS symptoms occur episodically with subsequent remission and, as the disease progresses, symptom-free intervals often become shorter. Depending on the organ system affected, the symptoms can vary and resemble those of systemic mastocytosis [8]. The MCAS symptoms may include sudden onset of tachycardia, hypotensive syncope, dizziness, flushing, urticaria, angioedema, pruritus, abdominal cramps, nausea, vomiting, diarrhea, rhinorrhea, sneezing, wheezing, impaired concentration, fatigue as well as inflammation of the mucosa of the gastrointestinal tract and the respiratory tract [8, 10]. The combination of several of these listed symptoms occurring in different organ systems indicates the presence of MCAS [10]. Gastrointestinal symptoms are frequently reported by these patients and are often mistaken by physicians as functional gastrointestinal disorders. This syndrome can be diagnosed by medical history and certain biomarkers and has so far never been described in the context of MALS [11, 12].

For the diagnosis of MALS, it is crucial to understand that radiological diagnosis of MALS does not correlate with MALS specific symptoms. Celiac artery stenosis is reported population-wide in up to 6.7% and remains asymptomatic in the vast majority of patients [13].

The European Society for Vascular Surgery (ESVS) clinical practice guidelines recommends dynamic duplex abdominal ultrasonography (DUS) as primary screening method to diagnose MALS. Stenosis is respiratory-dependent and becomes more obvious with deep expiration, accordingly respiratory maneuvers are needed for diagnosis. DUS-based diagnostic criteria have been defined in several studies and include peak systolic velocity (PSV) > 200 cm/s and end-diastolic velocity (EDV) > 55 cm/s [14, 15]. The classic ‘hook-like’ downward displacement followed by a dilatation of the celiac artery is also a typical finding [14]. CT angiography (CTA) in deep expiration with 3D reformatting is the gold standard of MALS diagnosis and has an excellent sensitivity and specificity of 96% and 94%, respectively [14, 16]. Furthermore, mesenteric ischemia can be detected by gastric exercise tonometry (GET) and gastroscopy assisted laser doppler flowmetry combined with visible light spectroscopy [17, 18].

Laparoscopic celiac artery decompression has become increasingly accepted as standard surgical management in MALS. This technique aims to restore normal celiac blood flow in symptomatic patients. Though surgical success rates vary widely between 50 and 86% [19, 20]. Some authors have even suggested that no DUS or CTA parameter are able to predict clinical response [21]. In addition to surgical treatment, percutaneous transluminal angioplasty (PTA) has proven as useful therapy after surgical decompression of the celiac artery, if flow abnormalities persist postoperatively. In comparison, success rate of solemn PTA therapy in MALS has been poor which might be explained by the sustained extrinsic pressure on the celiac artery [20].

Several authors have confirmed that adequate selection of patients is the most important factor to improve surgical treatment outcome in MALS. However, preclinical criteria to identify patients who are likely to benefit from surgery do not exist. Therefore, we wanted to analyze our single center experience in one of the largest MALS cohorts accompanied by long-term results in 20 patients. The primary aim of this study is to define preclinical parameters to distinguish patients that would benefit from surgical release.

## Materials and methods

### Diagnostic workup

20 patients diagnosed with MALS were retrospectively enrolled in this single-center study. All patients underwent surgery at the University Hospital Bonn between 2016 and 2021. Patients were routinely followed-up at least every year. The study was conducted in accordance with the Declaration of Helsinki Ethical Principles, Good Clinical Practices and approved by the hospitals data protection officer.

Standard clinical and diagnostic parameters were analyzed including age, gender, preoperative body mass index (BMI), comorbidities, duration of the procedure, length of hospital stay, perioperative complications. Besides, radiologic data like peak preoperative and postoperative celiac artery velocities on DUS during both inspiratory and expiratory phase were measured. Additionally, the narrowest diameter of the celiac artery was also recorded on CTA.

### Diagnostic evaluation

**Fig. 1:**
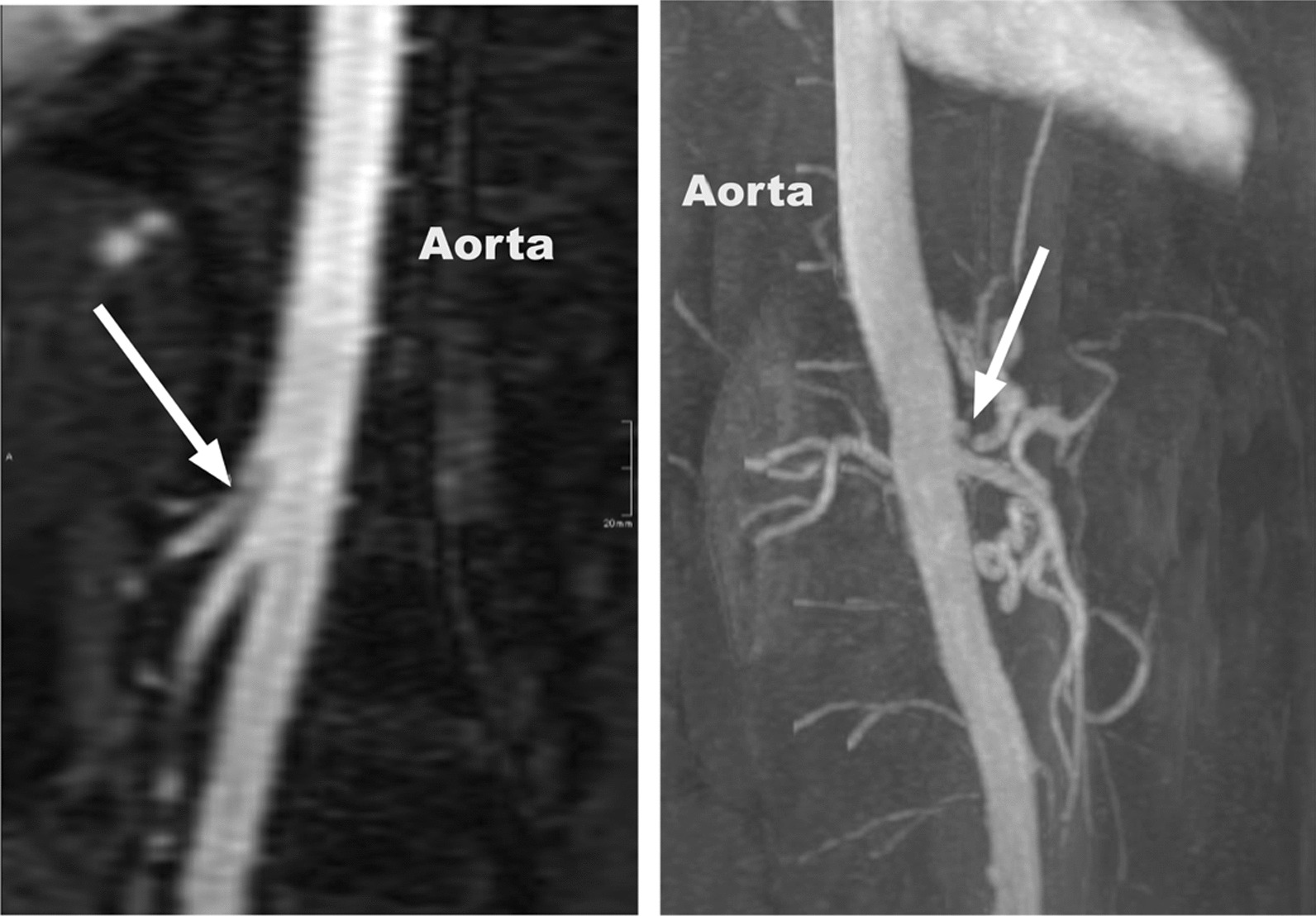
Computed tomography angiography (CTA) image (left) and 3D MRI reconstruction (right) showing celiac artery stenosis

All patients received a celiac axis DUS and CTA preoperatively and were postoperatively reevaluated with a second celiac axis DUS (Fig. 1). All ultrasound examinations were performed by board certified vascular surgeons.

In all patients, general gastrointestinal disorders were ruled out and the treatment was discussed in an interdisciplinary meeting with gastroenterologists, radiologists and surgeons. However, MCAS was not considered an exclusion criteria. DUS with peak celiac artery velocities > 200 cm/s was considered diagnostic for MALS. CTA criteria include specific imaging findings of celiac artery stenosis, such as focal celiac artery stenosis or demonstration of the characteristic hooklike morphology of the celiac artery.

### Operative technique

**Fig. 2:**
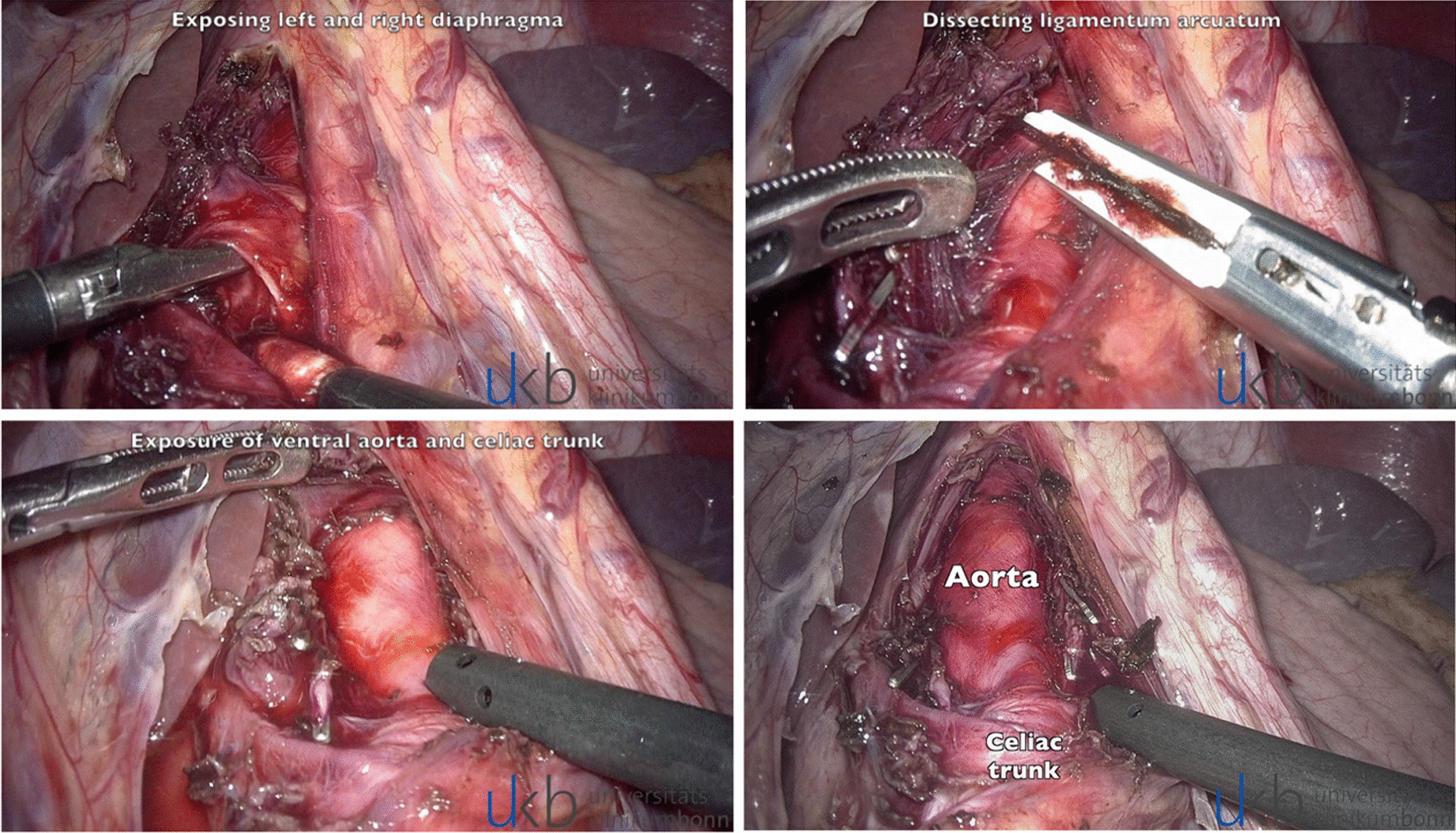
Surgical images. Exposing left and right diaphragm. Dissecting ligamentum arcuatum. Exposure of ventral aorta and celiac trunc

In the beginning of all operations, other intraabdominal pathologies were excluded by laparoscopy. The gastrohepatic ligament was divided to facilitate the identification of the right and left crus of the diaphragm. The stomach was retracted, exposing the anterior surface of the aorta. Following the superior aspect of the left gastric artery, the coeliac trunk was located. Next complete dissection of all tissue layers covering the anterior and mediolateral celiac artery (including large nerve complexes and lymphatic vessels) was then performed (Fig. 2). Treatment success was defined as a complete release of the celiac artery with no remaining stenosis in postoperative DUS, improvement in symptoms (at least 50% decrease in symptomatic pain episodes, measured by 0–10 numeric pain rating scale) and a decreased use of analgesics of at least 50%.

### Statistical analysis

Data was analyzed using SPSS version 28 (IBM Corp., IBM SPSS Statistics, Chicago, IL). We described the baseline characteristics for the overall population and different techniques attainment levels using mean values and standard deviations for continuous variables. Mann–Whitney U test was performed, where appropriate among groups two-sided p-values less than 0.05 were considered statistically significant.

## Results

### Patient characteristics

Mean age of patients (n = 20) was 43.6 years (range: 19–73) of which 75% were females. Patient characteristics are shown in Table 1. Two patients (10%) required conversion to open surgery due to intraoperative bleeding. The mean length of the operation was 143 min (SD: 36.3). The average length of hospitalization was 8.3 days (range 5–12 days). The origin of the celiac artery was visually free of external strictures and no remaining stenosis was observed in postoperative DUS in 90%. Two patients (10%) required postoperative PTA due to persistent flow abnormalities and symptoms. Two patients (10%) suffered from postoperative paralytic ileus. The mean follow-up time was 21.2 months (range: 9–46 months).

**Table 1:** Patient characteristics and postoperative outcome

	All groups	Symptom relief	Persistent symptoms	p value
Patients	20	12 (60%)	8 (40%)	
Age (mean)	38	38	37	0.938
Sex				1.0
Male	5 (25%)	3 (25%)	2 (25%)	
Female	15 (75%)	9 (75%)	6 (75%)	
BMI	21.1	21.9	19.8	0.063
Duration of symptoms before diagnosis (months)	45	29	68.9	0.054
Occlusion of arteria (%)	75	74	75	0.808
Operation				0.767
Laparoscopic	18 (90%)	11 (91.7%)	7 (87.5%)	
Open	2 (10%)	1 (8.3%)	1 (12.5%)	
Operation time (min)	162	153	174	0.563
Hospital stay (days)	8.3	8.25	8.4	0.938
Follow up time (months)	21.2	21.8	20.4	
Comorbidities				
Mast cell activation syndrome	7 (35%)	2 (18.2%)	5 (62.5%)	0.040
Cardiovascular risk factors	2 (10%)	1 (9.1%)	1 (12.5%)	

### Correlation with postoperative outcome

Out of 20 operations, 12 patients (60%) had a relief of their symptoms and simultaneous decrease of analgetic use, while 8 patients had persistent symptoms (less than 50% decrease in symptomatic pain episodes and less than 50% decrease of analgetic use).

No clinical variable predicted the postoperative symptom relief (Table 1) despite coexisting mast cell activation syndrome. Herein, MCAS correlated significantly with persistent symptoms after operation. Moreover, there was a trend that longer duration of symptoms before diagnosis and a lower BMI correlated with persistent symptoms (p = 0.054, p = 0.063), however it did not reach statistical significance. Moreover, the severity of stenosis on conventional imaging had no impact on treatment efficacy (Additional file 1: Table S1).

## Discussion

MALS is a rare disease (ORPHA: 293,208) that affects approximately 2 per 100,000 people, although the presence of significant celiac artery compression in cross sectional imaging is significantly higher and reaches up to 7% [22]. Due to its rarity, MALS remains understudied with missing large MALS trials. Only few studies with more than 20 patients exist and are displayed in Additional file 2: Table S2 [5, 23–28]. The vast majority of available literature consists of case studies. Given the nonspecific symptoms and the overlap with a wide range of gastrointestinal diseases, diagnosis is often delayed and made by exclusion of other pathologies. This delayed diagnosis may have a significant psychological and social impact on affected individuals. Preoperative patient selection is key for treatment success. However, no parameters identifying patients that will benefit most from surgical release have been defined yet. In one of the largest MALS cohorts (n = 20) with excellent follow-up we show that patients with coexisting mast cell activation syndrome are less likely to benefit from operative MAL release, while all other parameters failed to predict surgical outcome.

Mast cell disorders are conditions in which mast cells are either increased in number, hyper-reactive, or both. A key feature in patients with MCAS is recurrent episodes of systemic anaphylaxis with a variable clinical phenotype affecting multiple organs, such as cardiovascular, dermatologic, respiratory and gastrointestinal system [29]. High levels of mast cell mediators are released during those episodes and respond to treatment with inhibitors or blockers of mast cell mediators. Symptoms significantly overlap with MALS and are typically of gastrointestinal nature including diarrhea, nausea with vomiting and crampy abdominal pain. Moreover, patients often present fluctuating patterns of symptoms, which depend on the tissue responses to mast cell mediators released both spontaneously and in response to trigger stimuli. Therefore, even after a negative gastrointestinal check-up (including endoscopy) MCAS symptoms might be interpreted as MALS symptoms in the presence of celiac artery stenosis and surgical treatment should be evaluated carefully.

A hallmark of MACS is the proneness for multiple allergies. Our data suggest that in suspected MALS patients, a history of multiple allergies should trigger MACS exclusion. Ultimately, the cause of each patient’s symptoms and the response to treatment may vary, and selection of patients who are most likely to respond to surgical MAL release may best be accomplished through a constellation of clinical and imaging findings. The heterogeneity of the disease leads to the problem of a difficult and lengthy diagnostic process. The diagnosis has to be made primarily based on clinical findings and diagnostic criteria consisting of some laboratory parameters and immunohistochemical findings in biopsies. Currently, two approaches to diagnose MCAS are discussed, termed Consensus-1- and Consensus-2-criteria [10, 30]. Following the Consensus-2 criteria, up to 17% of the German population are suspected to suffer from MCAS [31]. Due to the lack of knowledge about etiology and pathology, in combination with often unspecific clinical presentation, MCAS patients suffer from delayed diagnosis and misdiagnoses, often for decades. Further delay in access to effective, quality-of-life-improving treatment should therefore be prevented at all cost.

In our series there is a predominance of women with 75%, which is consistent with the described 3:1 female to male ratio in most studies [32]. The mean patient age in this study is slightly above the described age, between 18 and 30 years, which might correlate to the long lead time to diagnosis [32, 33]. Interestingly, lead time to diagnosis also seems to influence surgical outcome. Herein, median duration of symptoms is 48 months and above the published median of 15 months (range 2–240) in other series [7]. This might be due to the fact that 35% of patients in this study presented with MCAS, a gastrointestinal disorder that is often present with apparent irritable bowel syndrome, dyspepsia and nausea, and is often diagnosed with significant delays [11, 12].

Our data show that the severity of stenosis on conventional imaging had no impact on treatment efficacy in accordance with recently published data [21]. This suggests that vascular compromise may not be the primary cause of pain in patients presenting with this syndrome and once again complicate patient selection for “successful” surgery. Future investigation incorporating the neurogenic basis of MALS pain, such as with diagnostic celiac ganglion blockade, would be helpful in further elucidating the enigmatic pathophysiologic process of MALS [5]. Moreover, we describe patients with persistent symptoms presented in trend with a longer duration of symptoms before MALS diagnosis. This might be due to neuropathic component that is suggested to lead to a chronic irritation of sympathetic pain fibers, splanchnic vasoconstriction and ischemia [5, 6].

Laparoscopic decompression was shown as an effective treatment for “true” MALS patients and can provide immediate symptomatic relief. Our series of patients experienced good outcomes, comparable to those reported in the literature. The laparoscopic approach is widely adopted owing to benefits such as shorter hospital stay, decreased time to feeding, smaller risk of postoperative complications, decreased blood loss and greater postoperative pain relief. Despite our single center experience and the treatment success, manipulation of tissue layer close to the celiac axis is associated with possible complications. Herein, we converted to an open approach due to intraoperative arterial bleeding in two patients.

These studies results are limited by its retrospective character conducted at a single institution and its small sample size. Nevertheless, our series is comparatively large with respect to the existing literature and reflects the low prevalence of MALS. Future registry-based studies are needed to compare long-term results of surgical celiac artery decompression and conservative therapy to confirm our findings and further define preclinical parameters that help to distinguish patients that would benefit from surgical release in order to develop evidence-based guidelines for the management of MALS.

## Conclusions

Overall, laparoscopic MAL release can provide immediate symptomatic relief. Selection of patients who are most likely to respond to surgical MAL release may best be accomplished through a constellation of clinical and imaging findings with an interdisciplinary team of gastroenterologists, radiologists and surgeons. In particular, MCAS should be considered and tested for in patients presenting with multiple allergies. Furthermore, surgical treatment should be evaluated carefully in patients with co-existing mast cell activation syndrome.

### Supplementary Information

**Additional file 1. Table S1**. Imaging findings in CTA and DU.

**Additional file 2. Table S2**. Studies on the surgical outcome of celiac artery decompression in median arcuate ligament syndrome (MALS), with patient size of more and less than 20 patients.

## Data Availability

The datasets generated and/or analyzed during the current study are not publicly available due to individual patient privacy and lack of consent but are available from the corresponding author on reasonable request.
