# Supplementary material for: Prognostic factors for the long term outcome after surgical celiac artery decompression in MALS
Source: Orphanet J Rare Dis. 2023 Oct 23;18:334. doi: 10.1186/s13023-023-02952-7 (PMC10594872; doi:10.1186/s13023-023-02952-7)
Supplement: Supplementary file 1 — Additional file 1. Table S1. Imaging findings in CTA and DU. [file 13023_2023_2952_MOESM1_ESM.pdf]

PATIENT #	Persistent symptoms	Minimal diameter of celiac artery (mm) in CTA	Preoperative Expiratory Vmax (cm/s)	Postoperative Expiratory Vmax (cm/s)
1	Yes	3.0	349	191
3	Yes	2.9	357	197
4	Yes	4.3	189	102
6	Yes	1.5	520	270
11	Yes	3.4	342	207
12	Yes	3.3	285	178
15	Yes	1.9	508	287
18	Yes	4.1	158	126
2	No	3.1	259	164
5	No	2.8	327	200
7	No	4.1	169	108
8	No	3.5	266	194
9	No	1.6	460	307
10	No	4.3	289	155
13	No	1.1	575	
14	No	3.4	272	187
16	No	2.7	421	183
17	No	3.0	476	348
19	No	1.9	267	179
20	No	4.4	228	145

Supplemental Table S1. Imaging findings in CTA and DUS
