# Supplementary material for: Prognostic factors for the long term outcome after surgical celiac artery decompression in MALS
Source: Orphanet J Rare Dis. 2023 Oct 23;18:334. doi: 10.1186/s13023-023-02952-7 (PMC10594872; doi:10.1186/s13023-023-02952-7)
Supplement: Supplementary file 2 — Additional file 2. Table S2. Studies on the surgical outcome of celiac artery decompression in median arcuate ligament syndrome (MALS), with patient size of more and less than 20 patients. [file 13023_2023_2952_MOESM2_ESM.pdf]

Reference	Number of patients	Mean follow-up duration (month)	Improvement of symptoms n (%)
Columbo, 2015	21	7	18(81)
Fernstrum, 2020	27	30	17 (68)
Ho, 2017	43	25	16 (37)
Khrucharoen, 2020	41	16	18 (44)
Pather, 2021	46	96	30 (65)
Van Petersen, 2009	42	20	41 (98)
Weber, 2016	31	52	20 (64)
Baccari, 2009	16	28	16 (100)
Baldassarre, 2007	1	3	1 (100)
Berard, 2012	11	35	8 (73)
Berge, 2020	12	18	7 (58)
Carbonell, 2005	1	7	1 (100)
Cienfuegos, 2017	13	117	9 (69)
DeAth, 2018	6	109	6 (100)
Do, 2013	12	22	8 (67)
Dordoni, 2002	1	6	1 (100)
Duffy, 2008	1	10	1 (100)
Fajer, 2014	1	3	1 (100)
Gander, 2010	1	4	1 (100)
Grus T, 2018	8	12	8 (100)
Jaik, 2008	1	6	1 (100)
Jarry, 2008	1	1	1 (100)
Joyce, 2013	6	13	6 (100)
Kafadar, 2021	10	6	10 (100)
Kohn, 2011	6	49	5 (83)
Lainez, 2013	1	0.5	1 (100)
Marable, 1968	17	0	13 (76)
Mihas, 1977	4	0	0 (0)
Muqeetadnan, 2013	1	0	1 (100)
Nguyen, 2012	5	20	5 (100)
Reddy, 2019	3	15	3 (100)
Relles, 2012	3	11	2 (67)
Roayaie, 2002	1	3	1 (100)
Rogers, 1982	7	58	2 (29)
Roseborough, 2009	15	44	14 (93)
Rotellar, 2009	7	6	3 (43)
Sahm, 2020	16	5	14 (87)
Sultan, 2013	11	60	8 (73)
Thoolen, 2015	9	6	4 (44)
Tulloch, 2010	14	14	8 (57)
Tsujimoto, 2012	1	0	1 (100)
Vaziri, 2009	3	6	3 (100)
Watson, 1977	18	30	14 (78)
Wani, 2012	2	2	2 (100)
You, 2013	1	0.5	1 (100)

Supplemental Table S2. Studies on the surgical outcome of celiac artery decompression in median arcuate ligament syndrome (MALS), with patient size of more and less than 20 patients.
